# Adolescent health outcomes: associations with child maltreatment and peer victimization

**DOI:** 10.1186/s12889-022-13310-w

**Published:** 2022-05-06

**Authors:** Samantha Salmon, Isabel Garcés Dávila, Tamara L. Taillieu, Ashley Stewart-Tufescu, Laura Duncan, Janique Fortier, Shannon Struck, Katholiki Georgiades, Harriet L. MacMillan, Melissa Kimber, Andrea Gonzalez, Tracie O. Afifi

**Affiliations:** 1 Department of Community Health Sciences, University of Manitoba, S113-750 Bannatyne Avenue, Winnipeg, MB R3E 0W5 Canada; 2 Faculty of Social Work, University of Manitoba, 173 Dafoe Road W., Winnipeg, MB R3T 2N2 Canada; 3 Department of Health Research Methods, Evidence & Impact, McMaster University, 1280 Main Street West, Hamilton, ON L8S 4L8 Canada; 4 Offord Centre for Child Studies, Department of Psychiatry & Behavioural Neurosciences, McMaster University, 1280 Main Street West, Hamilton, ON L8S 4L8 Canada; 5 Department of Pediatrics, McMaster University, 1280 Main Street West, Hamilton, ON L8S 4L8 Canada; 6 Department of Psychiatry, University of Manitoba, PZ433-771 Bannatyne Avenue, Winnipeg, MB R3E 3N4 Canada

**Keywords:** Child maltreatment, Peer victimization, Mental health, Non-suicidal self-injury, Suicidality, Physical health, Adolescents, Sex differences

## Abstract

**Background:**

Child maltreatment (CM) and peer victimization (PV) are serious issues affecting children and adolescents. Despite the interrelatedness of these exposures, few studies have investigated their co-occurrence and combined impact on health outcomes. The study objectives were to determine the overall and sex-specific prevalence of lifetime exposure to CM and past-month exposure to PV in adolescents, and the impact of CM and PV co-occurrence on non-suicidal self-injury, suicidality, mental health disorders, and physical health conditions.

**Methods:**

Adolescents aged 14–17 years (*n* = 2,910) from the 2014 Ontario Child Health Study were included. CM included physical, sexual, and emotional abuse, physical neglect, and exposure to intimate partner violence. PV included school-based, cyber, and discriminatory victimization. Logistic regression was used to compare prevalence by sex, examine independent associations and interaction effects in sex-stratified models and in the entire sample, and cumulative effects in the entire sample.

**Results:**

About 10% of the sample reported exposure to both CM and PV. Sex differences were as follows: females had increased odds of CM, self-injury, suicidality, and internalizing disorders, and males had greater odds of PV, externalizing disorders, and physical health conditions. Significant cumulative and interaction effects were found in the entire sample and interaction effects were found in sex-stratified models, indicating that the presence of both CM and PV magnifies the effect on self-injury and all suicide outcomes for females, and on suicidal ideation, suicide attempts, and mental health disorders for males.

**Conclusions:**

Experiencing both CM and PV substantially increases the odds of poor health outcomes among adolescents, and moderating relationships affect females and males differently. Continued research is needed to develop effective prevention strategies and to examine protective factors that may mitigate these adverse health outcomes, including potential sex differences.

**Supplementary Information:**

The online version contains supplementary material available at 10.1186/s12889-022-13310-w.

Child maltreatment (CM) and peer victimization (PV) are two forms of interpersonal victimization affecting children and adolescents. CM is defined by the World Health Organization as “the abuse and neglect that occurs to children under 18 years of age,” including “all types of physical and/or emotional ill-treatment, sexual abuse, neglect, negligence and commercial or other exploitation, which results in actual or potential harm to the child’s health, survival, development or dignity in the context of a relationship of responsibility, trust or power” [1]. CM commonly occurs in the home by parents or caregivers, but may also occur in other settings or with other perpetrators. CM is often operationalized in research as exposure to physical, sexual, or emotional abuse, physical or emotional neglect, or exposure to intimate partner violence (EIPV) during childhood. PV is defined as physical and non-physical forms of aggression among peers (i.e., children or adolescents of similar age, but not siblings). Although much of the literature is specific to bullying victimization, which falls within the domain of PV, PV is defined more broadly to overcome some of the limitations of the traditional conceptualization of bullying [2]. Extensive research has established CM as an important risk factor for a range of mental and physical health conditions [3–6], non-suicidal self-injury (NSSI) [7], and suicide ideation, attempts or death [8, 9]. Likewise, PV is a risk factor for the same outcomes [9–15]. Such experiences of victimization can have devastating consequences for the safety, health, and wellbeing of children and adolescents [3, 7–14], with sequelae that may persist into adulthood [4–6, 9, 15].

Since CM and PV are risk factors for the same outcomes, it is possible that the combined impact of exposure to both forms of victimization may have cumulative or interaction effects on mental and physical health. Cumulative effects are commonly examined in the childhood adversity literature using the cumulative risk model by summing a count of exposures into a cumulative risk index [16, 17]. A key strength of this approach is determining whether the joint effect of both exposures together is greater than the effect of each exposure considered separately. Specifically, individuals exposed to both types of victimization (both CM and PV) may have increased risk of poor outcomes compared to those not exposed or exposed to only one type of victimization (CM only or PV only). This is particularly important for informing public health strategies. If experiencing both CM and PV is indeed more harmful than CM or PV alone, then interventions targeting both may be more effective than those aimed at CM and PV separately [17]. However, a limitation of the cumulative risk index measured as a count variable is the inability to distinguish between different exposures combined into the same category (e.g., CM only and PV only), overlooking the possibility that different exposures may not have the same degree of risk for the outcome [17]. Instead, it is more informative to present the independent effects of each exposure alongside the joint effect. While the cumulative risk model examines joint effects on an additive scale, it is also possible that joint effects may occur on a multiplicative scale, determined by the statistical significance of an interaction term between CM and PV [18, 19]. Specifically, the associations between CM and mental and physical health may depend on whether the individual also experienced PV, in a way that is not simply additive. Consistent with the ecological theory of development, which postulates that a child’s development is influenced by different ecological contexts (e.g., family, school, and peers) that interact with one another [20], it is possible that the effect of exposure to one form of victimization is moderated in the context of the other. It may be the case that victimization experienced across different ecological contexts increases the risk of adverse outcomes due to an absence of safe environments that may mitigate some of the harmful effects. Importantly, cumulative effects may be observed even in the absence of interaction effects; it is therefore recommended that both cumulative and interaction effects are examined [18, 19].

To date, few studies have examined CM and PV co-occurrence. In an adolescent sample, Afifi and colleagues (2020) assessed cumulative and interaction effects between exposure to any adverse childhood experiences (ACEs), which included three types of CM (emotional abuse, emotional neglect, and EIPV), and exposure to PV on cigarette, vaping, alcohol, and cannabis use [21]. Interaction effects were examined with an interaction term between ACEs and PV, whereas cumulative effects were assessed by computing a four-level mutually-exclusive variable to discern the effects of exposure to ACEs only, PV only, and the joint effect of both ACEs and PV, as compared to adolescents with no exposure [21]. Cumulative effects were found indicating that adolescents exposed to both ACEs and PV had greater odds of substance use compared to adolescents with no exposure as well as compared to those who experienced ACEs only, but there was no evidence of interaction effects [21]. Similarly, Lereya et al. (2015) examined data from two longitudinal studies and observed significant cumulative effects indicating that experiencing both CM and bullying victimization compared to no exposure was associated with increased odds of mental health outcomes in early adulthood, including anxiety, depression, and self-harm or suicidal ideation, plans, or attempts [22]. Furthermore, Sansen and colleagues (2014) tested the interaction between CM and relational PV (e.g., social exclusion) and found a significant moderating effect on psychopathology for the self-selected community sample in their study, but did not observe significant interactions for the clinical or student samples [23]. In another recent study, Tremblay-Perreault and Hébert (2020) observed cumulative effects between child sexual abuse and PV in associations with both internalizing and externalizing behaviour problems in a pediatric sample, but did not test interactions [24]. Overall, the current literature provides initial support for cumulative effects of CM and PV co-occurrence, but there is limited evidence of interaction effects.

Previous studies are also limited by the absence of an examination of sex differences in the impact of co-occurring CM and PV. Interventions may require tailored approaches for females and males. Sex differences in the overall prevalence of CM and PV depend, in part, on specific victimization types included in its measurement. For example, sexual abuse has consistently been shown to be more common in females, and some studies have shown physical abuse to be more common in males [25, 26]. A recent systematic review also reported higher prevalence of emotional abuse and neglect for females, though differences were not statistically tested [27]. In the PV literature, physical PV types are more prevalent in males, while social and cyber PV are more common in females [28, 29]. There is also limited evidence of possible sex differences in the effects of CM and PV on health outcomes. For example, pooled meta-analytic results showed stronger effects in the associations between CM and internalizing problems for adult females, though sex differences were not statistically significant potentially due to the limited number of eligible studies and lack of statistical power [30]. In adolescents, Wei et al. (2021) found greater associations between individual CM types and depressive symptoms in females compared to males [31]. Similarly, Hagborg et al. (2017) found that associations between emotional neglect and internalizing symptoms were magnified in female compared to male adolescents [32]. Furthermore, a recent study reported that social and cyberbullying had stronger associations with emotional problems for females, whereas cyberbullying had stronger associations with behavioural problems for males [29]. It is therefore possible that cumulative or interaction effects in the associations between CM, PV, and mental and physical health differ by sex.

The objectives of the current study were to determine: 1) the prevalence of CM and PV co-occurrence among adolescents aged 14 to 17 years in Ontario, Canada, 2) whether prevalence differs for males and females, and 3) the interaction and cumulative effects of co-occurring CM and PV on NSSI, suicidal ideation, plans and attempts, internalizing and externalizing mental health disorders, and physical health conditions in the total sample and sex-stratified models after adjusting for sociodemographic characteristics.

## Methods

### Data and sample

The current study involved a sample of adolescents from the provincially-representative, cross-sectional 2014 Ontario Child Health Study (OCHS) [33]. This study of children aged four to 17 years was conducted in Ontario, Canada; questionnaires were administered by Statistics Canada. In total, 10,802 children from 6,537 households participated (response = 50.8%) [33]. The sample for this study was restricted to a subset of adolescents aged 14 to 17 years, including the selected child and their sibling(s), who completed individual questionnaires on a laptop (*n* = 2,910). Ethics approval for the original survey was granted by the Hamilton Integrated Research Ethics Board at McMaster University. Further detail on the methods of the 2014 OCHS has been reported previously [33].

### Measures

#### Child maltreatment

Exposure to child maltreatment included the measurement of physical abuse, sexual abuse, emotional abuse, physical neglect, and EIPV. Physical abuse, sexual abuse, and EIPV were assessed with items adapted from the Childhood Experiences of Violence Questionnaire (CEVQ), which produces valid and reliable scores [34], while emotional abuse and physical neglect items were obtained from the National Longitudinal Study of Adolescent to Adult Health [35]. For each item, respondents were prompted to think about things that may have happened “at any time while growing up.” Physical abuse was assessed with three items asking how many times they were (a) slapped on the face, head or ears or hit or spanked with something hard by an adult, (b) pushed, grabbed, shoved, or had something thrown at them by an adult, or (c) kicked, bit, punched, burnt, or physically attacked by an adult. Sexual abuse was assessed with two items asking how many times an adult (a) forced or attempted to force the respondent into any unwanted sexual activity with threats or physical violence, or (b) touched the respondent against their will in any sexual way. Emotional abuse was assessed with one item asking how many times parents/caregivers said things that hurt the respondent’s feelings or made them feel like they were not wanted or loved. Physical neglect was assessed with one item asking how many times parents/caregivers did not take care of the respondent’s basic needs (e.g., keeping them clean, providing food or clothing). Finally, EIPV was assessed with two items asking how many times the respondent saw or heard parents/caregivers (a) say hurtful or mean things to each other or another adult in the home or (b) hit each other or another adult in the home. Response options for each item were: “Never,” “1–2 times,” “3–5 times,” “6–10 times,” and “More than 10 times.” Each CM type was coded separately based on previously used cut-points, which varied depending on the severity and frequency of each item [34]. Specifically, physical abuse required a response of three or more times to either one or both of the first two items and/or a response of at least one time to the third item; sexual abuse required a response of at least one time to either one or both items; emotional abuse required a response of six or more times to the single item; physical neglect required a response of at least one time to the single item; and EIPV required a response of six or more times to the first item and/or three or more times to the second item. Finally, the five CM types were subsequently combined into a dichotomous measure of any lifetime CM.

#### Peer victimization

PV was measured using the School Crime Supplement of the National Crime Victimization Survey [36]. Respondents that attended school for at least one month since September 2014 were asked how often during the present school year another student: “made fun of you, called you names or insulted you,” “spread rumours about you,” “threatened you with harm,” “pushed you, shoved you, tripped you, or spit on you,” “tried to make you do things you did not want to do, for example, give them money or other things,” “excluded you from activities on purpose,” “destroyed your property on purpose,” “posted hurtful information about you on the Internet,” “threatened or insulted you through email, instant messaging, text messaging, or an online game,” “purposefully excluded you from an online community,” or “called you an insulting or bad name at school having to do with your race, religion, ethnic background or national origin,” “…any disability you may have,” or “…your sexual orientation.” Although not often included, recent research has shown that discriminatory PV is common among adolescents [28] and is associated with poorer mental health [37]. Response options for each item were: “Never,” “Once or twice this school year,” “Once or twice this month,” “Once or twice this week,” and “Almost every day.” Consistent with past research, responses were dichotomized as “once or twice this month” or more often versus “never” or “once or twice this school year” [38]. All items were then combined into a dichotomous measure of any past-month PV.

#### Cumulative exposure

The two dichotomous variables for lifetime exposure to CM and past-month exposure to PV were summed into a cumulative exposure variable. However, rather than simply examining a count of exposures (0, 1, 2), we separated those who reported exposure to CM only versus PV only resulting in a categorical variable with four mutually exclusive levels: no CM or PV, CM only, PV only, and both CM and PV.

#### Non-suicidal self-injury and suicidality

Adolescents were asked about NSSI and suicidal ideation with the questions: “In the past 12 months, did you ever deliberately harm yourself but not mean to take your life?” and “In the past 12 months, did you ever seriously consider taking your own life or killing yourself?”. Response options were “yes” or “no.” Those who responded affirmatively to the latter item for suicidal ideation were then asked about past-year suicidal plans and attempts with the questions: “In the past 12 months, did you make a plan about how you would take your own life or kill yourself?” (response options: “yes” or “no”) and “How many times did you actually try to take your own life or kill yourself?”, which included the response options “Never,” “Once,” and “More than once” and were coded as “once or more” versus “never” due to limited cell sizes.

#### Mental health disorders

The 2014 OCHS Emotional Behavioural Scales (OCHS-EBS) checklist, which has demonstrated validity and reliability [39], assessed six mental health disorders: generalized anxiety disorder (GAD), separation anxiety disorder (SAD), social phobia (SP), major depressive disorder (MDD), oppositional defiant disorder (ODD), and conduct disorder (CD). Adolescents were asked to self-report symptoms for each disorder experienced within the past six months (e.g., “I worry a lot.”) with the response options: “Never or not true,” “Sometimes or somewhat true,” and “Often or very true.” Responses were assigned a score from zero to two, respectively, and summed into an overall score for each disorder (with symptoms of GAD, SAD, and SP combined into any anxiety disorder). Using an existing approach to create binary classifications [39], each score was dichotomized using cut-points informed by global prevalence estimates: any anxiety disorder (6.5%), MDD (2.6%), ODD (3.6%), and CD (2.1%) [40]. Anxiety and MDD were combined into a single variable indicating the presence of one or both internalizing disorders and ODD and CD were combined into a single variable indicating the presence of one or both externalizing disorders. Finally, internalizing and externalizing disorders were combined into a dichotomous variable of any mental health disorder.

#### Physical health conditions

Adolescent self-reported, long-term physical health conditions diagnosed by a health professional included allergies, bronchitis, diabetes, heart condition/disease, epilepsy, cerebral palsy, kidney condition/disease, asthma, or any other long-term condition. A single dichotomous indicator of any physical health condition was created.

#### Covariates

Adolescent sex (male, female), age (14–17 years), ethnicity (white, non-white/multi-ethnicity), parent/caregiver-reported household income (less than $25,000, $25,000-$49,999, $50,000-$74,999, $75,000-$99,999, $100,000 or greater), single-parent household status (yes, no) based on demographic information collected from the parent/caregiver, and urbanicity (large urban, small-medium urban, and rural) based on current census population counts were included.

### Data analysis

First, sociodemographic characteristics describing the sample were computed. Second, weighted prevalence estimates of CM, PV, and each outcome were computed for the total sample and by sex. Sex differences were tested with unadjusted logistic regression analysis with males as the reference group. Third, the prevalence of each outcome by CM and PV exposure was computed, stratified by sex. Fourth, a series of nested sequential logistic regression models adjusting for sociodemographic characteristics (i.e., age, ethnicity, household income, single-parent household, and urbanicity) were conducted to assess independent associations and interaction effects between CM and PV with each outcome stratified by sex and in the total sample. Model 1 assessed CM, model 2 assessed PV, model 3 included both CM and PV, and model 4 tested the interaction between CM and PV. Models with statistically significant interaction terms were subsequently examined using plots of prevalence data for each outcome variable by presence or absence of CM and stratified by presence or absence of PV. Last, cumulative effects were examined by testing the association between the four-level mutually exclusive CM/PV variable (no CM or PV, CM only, PV only, both CM and PV) and each outcome using logistic regression adjusting for all covariates (including sex) in the entire sample with no CM or PV exposure as the reference group. Differences between each exposure category were then examined by sequentially changing the reference category in each regression model. Upon examination of the data, it was determined that due to small cell sizes, cumulative effects stratified by sex could not be examined. Bootstrap weights (Fay adjustment: 0.8) computed by Statistics Canada were applied to all analyses to ensure results were representative of the target population and to produce valid variance estimates. Statistical significance was set at *p* < 0.05.

## Results

Table 1 shows sociodemographic characteristics for the sample. Adolescents were evenly distributed across age (14 to 17 years) and sex (51.4% male). Most were white (60.5%) and residing in a two-parent household (76.1%) and large urban community (69.7%). Household income varied in distribution: 7.5% had a household income less than $25,000, 10.6% between $25,000 to $49,999, 22.7% between $50,000 to $74,999, whereas most (59.1%) had a household income of $75,000 or greater. Prevalence estimates of adolescent-reported CM, PV, and the health outcomes are provided in Table 2, with comparisons between females and males. Sex differences were found across estimates. The odds of experiencing CM, NSSI, suicidal ideation, plans, and attempts, and any internalizing mental health disorder were greater among females compared to males; whereas, the odds of experiencing PV (alone and in combination with CM), any externalizing mental health disorder, and any physical health condition were lower among females compared to males. Table 3 displays the prevalence of mental and physical health problems stratified by sex and CM/PV exposure. Among adolescents that reported past-month PV exposure, 53.0% of females and 46.5% of males reported also experiencing lifetimes CM exposure.

**Table 1:** Weighted prevalence of sample characteristics

Characteristic	Total Sample % (95% CI)
**Sex**	
Male	51.4 (51.4, 51.4)
Female	48.6 (48.6, 48.6)
**Age, years**	
14	23.8 (23.7, 23.8)
15	24.7 (24.7, 24.8)
16	25.1 (25.1, 25.2)
17	26.4 (26.3, 26.4)
**Ethnicity**	
White	60.5 (59.6, 61.5)
Non-white/multi-ethnicity	39.5 (38.5, 40.4)
**Household Income, $**	
Less than 25,000	7.5 (7.3, 7.8)
25,000 to 49,999	10.6 (10.3, 10.9)
50,000 to 74,999	22.7 (22.2, 23.3)
75,000 to 99,999	22.4 (21.7, 23.1)
100,000 or greater	36.7 (36.1, 37.3)
**Single Parent Household**	
No	76.1 (75.4, 76.7)
Yes	23.9 (23.3, 24.6)
**Urbanicity**	
Rural	14.0 (13.4, 14.8)
Small to medium urban	16.2 (15.0, 17.5)
Large urban	69.7 (68.5, 71.0)

*Abbreviations: CI* Confidence Interval

**Table 2:** Weighted prevalence of CM, PV, NSSI, suicidality, mental health disorders, and physical health conditions in the entire sample and stratified by sex

	Total Sample % (95% CI)	Females % (95% CI)	Males % (95% CI)	OR^a^ (95% CI)
Any CM^b^	26.4 (25.8, 27.0)	29.1 (28.3, 29.9)	23.9 (23.1, 24.7)	1.31 (1.24, 1.38)
Any PV^b^	20.3 (19.8, 20.9)	17.5 (16.9, 18.1)	22.9 (22.1, 23.8)	0.71 (0.67, 0.76)
**Co-occurrence**				
No CM or PV, ref	64.1 (63.3, 64.8)	63.5 (62.6, 64.3)	64.6 (63.6, 65.7)	1.00
CM Only	15.6 (15.1, 16.1)	18.9 (18.2, 19.7)	12.5 (11.8, 13.1)	1.55 (1.44, 1.66)
PV Only	10.3 (9.9, 10.7)	8.3 (7.8, 8.8)	12.3 (11.7, 12.9)	0.69 (0.63, 0.75)
CM & PV	10.0 (9.6, 10.5)	9.3 (8.9, 9.8)	10.6 (9.9, 11.4)	0.89 (0.82, 0.98)
**NSSI and Suicidality**^b^				
NSSI	9.3 (8.9, 9.7)	14.3 (13.7, 14.8)	4.5 (4.1, 5.0)	3.50 (3.15, 3.90)
Suicidal Ideation	8.6 (8.3, 9.0)	11.1 (10.6, 11.7)	6.3 (5.9, 6.7)	1.87 (1.72, 2.03)
Suicidal Plans	4.1 (3.9, 4.4)	4.8 (4.5, 5.2)	3.4 (3.2, 3.8)	1.42 (1.26, 1.60)
Suicide Attempts	4.6 (4.4, 4.9)	5.6 (5.2, 6.1)	3.7 (3.4, 4.0)	1.56 (1.36, 1.77)
**Mental Health**^b^				
Any Internalizing Disorder	6.5 (6.2, 6.8)	8.9 (8.4, 9.4)	4.2 (3.8, 4.7)	2.21 (1.94, 2.51)
Any Externalizing Disorder	3.7 (3.5, 4.0)	3.4 (3.1, 3.7)	4.1 (3.8, 4.4)	0.81 (0.72, 0.91)
Any Mental Health Disorder	9.0 (8.6, 9.3)	10.5 (10.0, 11.0)	7.5 (7.0, 8.0)	1.45 (1.33, 1.58)
**Physical Health**^b^				
Any Physical Health Condition	33.8 (33.2, 34.4)	32.6 (31.7, 33.6)	35.0 (34.1, 35.8)	0.90 (0.85, 0.95)

*Abbreviations: CI* Confidence Interval, *CM* Child Maltreatment, *NSSI* Non-suicidal Self-injury, *OR* Odds Ratio, *PV* Peer Victimization, *ref* reference category

^a^ Reference category is males

^b^ Reference category is no exposure

**Table 3:** Weighted prevalence of CM by PV and of NSSI, suicidality, mental health disorders, and physical health conditions by CM and PV stratified by sex

	Female Adolescents				Male Adolescents			
	CM	No CM	PV	No PV	CM	No CM	PV	No PV
	% (95% CI)	% (95% CI)	% (95% CI)	% (95% CI)	% (95% CI)	% (95% CI)	% (95% CI)	% (95% CI)
**Child Maltreatment**								
CM	–	–	53.0 (51.0, 55.0)	23.0 (22.1, 23.8)	–	–	46.5 (44.2, 48.8)	16.2 (15.3, 17.0)
No CM	–	–	47.0 (45.0, 49.0)	77.0 (76.2, 77.9)	–	–	53.5 (51.2, 55.8)	83.8 (83.0, 84.7)
**NSSI and Suicidality**								
NSSI	26.2 (24.9, 27.5)	9.9 (9.3, 10.5)	29.1 (27.2, 30.9)	10.5 (10.0, 11.1)	8.9 (7.8, 10.1)	2.5 (2.2, 2.9)	8.0 (6.9, 9.1)	2.6 (2.2, 2.9)
Suicidal Ideation	24.3 (22.9, 25.7)	6.2 (5.7, 6.7)	25.5 (23.7, 27.4)	7.6 (7.1, 8.1)	15.0 (13.8, 16.4)	3.4 (3.1, 3.7)	11.5 (10.3, 12.8)	3.5 (3.2, 3.9)
Suicidal Plans	11.8 (10.9, 12.7)	2.2 (1.9, 2.5)	13.7 (12.3, 15.3)	3.1 (2.7, 3.4)	6.9 (6.1, 7.9)	2.1 (1.8, 2.3)	5.6 (4.8, 6.4)	1.5 (1.3, 1.6)
Suicide Attempts	13.9 (12.8, 15.1)	2.4 (2.1, 2.8)	14.4 (12.9, 15.9)	3.3 (3.0, 3.7)	10.3 (9.0, 11.6)	1.8 (1.6, 2.1)	7.0 (6.0, 8.2)	1.9 (1.7, 2.2)
**Mental Health**								
Any Internalizing Disorder	16.9 (15.7, 18.1)	5.4 (5.0, 5.9)	25.4 (23.7, 27.3)	4.9 (4.5, 5.3)	8.2 (7.1, 9.4)	3.2 (2.7, 3.7)	13.2 (11.5, 15.1)	1.5 (1.3, 1.8)
Any Externalizing Disorder	9.3 (8.4, 10.3)	1.2 (1.0, 1.3)	14.3 (12.6, 16.0)	1.3 (1.2, 1.5)	7.8 (6.8, 8.9)	3.0 (2.7, 3.3)	9.3 (8.2, 10.5)	2.1 (1.9, 2.4)
Any Mental Health Disorder	20.7 (19.5, 22.0)	6.3 (5.8, 6.8)	31.6 (29.6, 33.7)	5.7 (5.3, 6.2)	15.0 (13.6, 16.5)	5.3 (4.8, 5.9)	20.8 (19.0, 22.8)	3.0 (2.8, 3.3)
**Physical Health**								
Any Physical Health Condition	43.2 (41.4, 45.1)	26.2 (25.3, 27.2)	34.2 (32.2, 36.2)	28.8 (27.9, 29.7)	33.2 (31.5, 35.0)	35.3 (34.4, 36.2)	37.9 (35.7, 40.1)	33.2 (32.2, 34.3)

*Abbreviations: CI* Confidence Interval, *CM* Child Maltreatment, *NSSI* Non-suicidal Self-injury, *PV* Peer Victimization

Independent associations and interaction effects between lifetime CM and past-month PV exposure with each outcome, in sex-stratified models and in the total sample, are presented in Table 4. In models adjusting for age, ethnicity, household income, single-parent household, and urbanicity, lifetime exposure to CM was found to be associated with increased odds of all outcomes in the total sample and for females and all except physical health conditions for males (Model 1). Exposure to past-month PV was found to be associated with increased odds of all outcomes for both females and males after adjusting for sociodemographic characteristics (Model 2). Results from Model 3 demonstrated that in the total sample and among female adolescents, CM remained significantly associated with all outcomes over and above PV, and that PV also remained significant with all outcomes over and above CM. Among male adolescents, with the exception of physical health conditions, CM exposure remained significantly associated with all other outcomes over and above PV, and PV also remained significant over and above CM in all other fully adjusted models (Model 3).

**Table 4:** Independent and interaction effects of CM and PV on NSSI, suicidality, mental health disorders, and physical health conditions stratified by sex and in the total sample

	NSSI	Suicidal Ideation	Suicidal Plans	Suicide Attempts	Any Internalizing Disorder	Any Externalizing Disorder	Any MH Disorder	Any PH Condition
	AOR (95% CI)	AOR (95% CI)	AOR (95% CI)	AOR (95% CI)	AOR (95% CI)	AOR (95% CI)	AOR (95% CI)	AOR (95% CI)
**Female Adolescents**								
Model 1: Any CM	3.53 (3.22, 3.87)	5.50 (4.93, 6.15)	6.41 (5.50, 7.46)	6.70 (5.69, 7.89)	3.97 (3.47, 4.53)	9.65 (8.04, 11.57)	4.43 (3.91, 5.03)	2.24 (2.03, 2.47)
Model 2: Any PV	3.22 (2.87, 3.60)	4.49 (3.91, 5.15)	4.54 (3.73, 5.53)	4.94 (4.07, 5.99)	8.27 (7.12, 9.60)	12.52 (10.17, 15.41)	8.86 (7.69, 10.21)	1.26 (1.13, 1.39)
Model 3: Any CM	2.66 (2.41, 2.92)	3.94 (3.51, 4.41)	4.96 (4.23, 5.82)	4.89 (4.17, 5.74)	2.57 (2.22, 2.98)	6.30 (5.21, 7.63)	2.99 (2.62, 3.43)	1.87 (1.70, 2.07)
Any PV	2.57 (2.27, 2.91)	3.14 (2.72, 3.63)	3.03 (2.45, 3.76)	3.29 (2.69, 4.02)	6.46 (5.54, 7.53)	7.54 (6.17, 9.21)	6.75 (5.87, 7.77)	1.12 (1.01, 1.25)
Model 4: CM x PV Interaction *p*-value	< .001	.006	< .001	< .001	.469	.570	.978	< .001
**Male Adolescents**								
Model 1: Any CM	2.86 (2.30, 3.55)	5.18 (4.46, 6.03)	3.77 (3.08, 4.62)	5.02 (4.10, 6.15)	3.13 (2.51, 3.90)	3.58 (3.01, 4.26)	4.03 (3.42, 4.75)	0.94 (0.85, 1.04)
Model 2: Any PV	2.62 (2.19, 3.14)	2.98 (2.47, 3.59)	3.33 (2.64, 4.19)	2.87 (2.19, 3.77)	11.42 (8.87, 14.70)	5.25 (4.31, 6.39)	11.08 (9.24, 13.28)	1.18 (1.04, 1.34)
Model 3: Any CM	1.95 (1.56, 2.45)	4.23 (3.55, 5.03)	2.82 (2.18, 3.65)	4.09 (3.26, 5.15)	2.01 (1.63, 2.49)	1.74 (1.41, 2.16)	2.06 (1.75, 2.43)	1.10 (1.00, 1.23)
Any PV	1.72 (1.38, 2.14)	2.19 (1.81, 2.66)	2.70 (2.06, 3.55)	2.06 (1.56, 2.72)	9.84 (7.62, 12.71)	4.58 (3.80, 5.53)	9.37 (7.83, 11.22)	1.12 (0.99, 1.27)
Model 4: CM x PV Interaction *p*-value	.545	< .001	NR	.003	.025	< .001	.578	.527
**Total Sample**								
Model 1: Any CM	3.62 (3.32, 3.95)	5.61 (5.09, 6.18)	5.33 (4.70, 6.04)	6.95 (6.04, 7.99)	3.71 (3.27, 4.20)	5.21 (4.56, 5.96)	4.15 (3.71, 4.64)	1.49 (1.39, 1.60)
Model 2: Any PV	3.06 (2.77, 3.37)	3.82 (3.42, 4.27)	4.30 (3.72, 4.98)	4.16 (3.55, 4.87)	8.81 (7.73, 10.03)	8.24 (7.09, 9.57)	9.34 (8.35, 10.45)	1.22 (1.12, 1.32)
Model 3: Any CM	2.60 (2.39, 2.84)	4.18 (3.78, 4.63)	4.06 (3.54, 4.67)	5.06 (4.36, 5.86)	2.20 (1.92, 2.52)	3.01 (2.64, 3.43)	2.36 (2.09, 2.65)	1.46 (1.36, 1.56)
Any PV	2.32 (2.08, 2.59)	2.72 (2.42, 3.06)	2.96 (2.52, 3.47)	2.77 (2.36, 3.26)	7.18 (6.24, 8.26)	6.09 (5.26, 7.06)	7.54 (6.70, 8.48)	1.11 (1.03, 1.21)
Model 4: CM x PV Interaction *p*-value	.012	< .001	< .001	< .001	< .001	.001	.165	< .001

*Abbreviations: AOR* Odds Ratio adjusted for age, ethnicity, household income, single-parent household, and urbanicity, *CI* Confidence Interval, *CM* Child Maltreatment, *MH* Mental Health, *NSSI* Non-suicidal Self-injury, *PH* Physical Health, *PV* Peer Victimization, *NR* Not Reported due to low cell counts

**Fig. 1:**
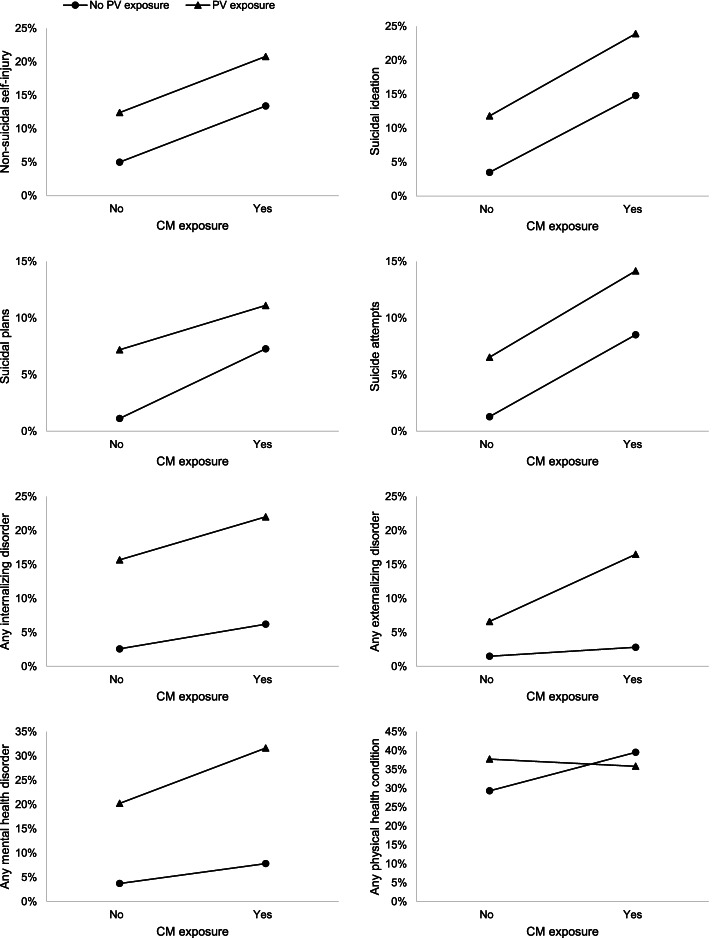
Prevalence of each outcome by child maltreatment exposure and stratified by peer victimization exposure in the total sample

In the total sample, significant interaction terms were found in Model 4 for all outcomes except any mental health disorder. Using plotted data, the relationships between CM and each outcome were moderated in the presence of PV (Fig. 1). Specifically, for NSSI, all suicide outcomes, and internalizing and externalizing mental health disorders, the relationships with CM were elevated for those with a history of PV. However, PV moderated the association between CM and physical health conditions in a different way; the relationship between CM and physical health conditions was slightly elevated for those with no PV history.

Among females, significant interaction terms were found in Model 4 for NSSI, suicidal ideation, suicidal plans, suicide attempts, and physical health conditions. Using plotted data (plots not shown), the associations between CM and NSSI, suicidal ideation, suicidal plans, and suicide attempts were moderated (worsened, steeper slopes) if PV was also present. However, the association between CM and physical health was slightly elevated among those without PV. Among males, significant interaction effects between CM and PV (Model 4) were found for suicidal ideation, suicide attempts, and internalizing and externalizing mental health disorders. The interaction effect for suicidal plans was not reported due to limited statistical power. Using plotted data (plots not shown), the relationships between CM and suicidal ideation and attempts were slightly elevated for those with a PV history. The relationship between CM and internalizing mental health disorders was moderated (worsened) if PV was also present, but was also high for those with PV and without CM. PV moderated the relationship differently for CM and externalizing mental health. Compared to those without CM, those with CM and PV histories had more elevated externalizing mental health problems, while those with CM and without PV histories had decreased externalizing mental health problems. Full Model 4 results (coefficients and confidence intervals) are provided in Supplementary Table 1.

Cumulative effects of CM and PV co-occurrence were examined among the entire sample (Table 5). Compared to adolescents with no exposure to CM or PV, exposure to CM only, PV only, or both CM and PV were all associated with increased odds of all outcomes after adjusting for covariates, including sex. Furthermore, after sequentially changing the reference category of the exposure it was determined that compared to experiencing CM only or PV only, exposure to both CM and PV was associated with greater odds of all outcomes except physical health conditions.

**Table 5:** Cumulative effects of CM and PV co-occurrence on NSSI, suicidality, mental health disorders, and physical health conditions in the entire sample

	NSSI	Suicidal Ideation	Suicidal Plans	Suicide Attempts	Any Internalizing Disorder	Any Externalizing Disorder	Any MH Disorder	Any PH Condition
	AOR (95% CI)	AOR (95% CI)	AOR (95% CI)	AOR (95% CI)	AOR (95% CI)	AOR (95% CI)	AOR (95% CI)	AOR (95% CI)
No CM or PV, ref	1.00	1.00	1.00	1.00	1.00	1.00	1.00	1.00
CM Only	2.84 ^a^ (2.55, 3.17)	4.97 ^a^ (4.38, 5.63)	7.43 ^a^ (6.07, 9.10)	6.96 ^a^ (5.68, 8.53)	2.82 ^a^ (2.36, 3.37)	2.33 ^a^ (1.97, 2.76)	2.55 ^a^ (2.20, 2.95)	1.68 ^a^ (1.56, 1.82)
PV Only	2.66 ^a^ (2.29, 3.09)	3.55 ^b^ (3.06, 4.13)	6.94 ^a^ (5.43, 8.87)	4.59 ^b^ (3.53, 5.98)	8.77 ^b^ (7.37, 10.44)	4.83 ^b^ (3.84, 6.06)	8.03 ^b^ (6.93, 9.30)	1.37 ^b^ (1.25, 1.50)
Both CM and PV	5.83 ^b^ (5.07, 6.71)	11.05 ^c^ (9.48, 12.88)	12.61 ^b^ (10.03, 15.85)	14.72 ^c^ (11.74, 18.47)	15.77 ^c^ (13.39, 18.57)	17.60 ^c^ (14.81, 20.91)	17.75 ^c^ (15.40, 20.46)	1.40 ^b^ (1.24, 1.57)

*Abbreviations: AOR* Odds Ratio adjusted for sex, age, ethnicity, household income, single-parent household, and urbanicity, *CI* Confidence Interval, *CM* Child Maltreatment, *MH* Mental Health, *NSSI* Non-suicidal Self-injury, *PH* Physical Health, *PV* Peer Victimization, *ref* reference category

^a,^^b,c^ AORs with different superscripts differ significantly at *p* < .05

## Discussion

The findings from this study examining CM and PV co-occurrence add to our understanding about their associations with mental and physical health problems for adolescents. In a provincially-representative sample of adolescents aged 14 to 17 years, it was found that over 35% have experienced CM and/or PV, and 10% have experienced both. Cumulative effects were found indicating that adolescents who experienced both CM and PV had substantially increased odds of NSSI, suicidality, and mental health disorders, but not physical health conditions. These findings are consistent with prior research reporting cumulative effects between CM and PV on anxiety, depression, NSSI, and suicidality in early adulthood [22], and between ACEs and PV on adolescent substance use [21]. In the current study, experiencing CM and PV together was also associated with significantly increased odds of all outcomes (except physical health conditions) compared to experiencing CM alone and compared to experiencing PV alone. This is of particular public health importance, indicating that strategies aimed at preventing NSSI, suicidality, and mental health disorders in adolescents should be multifaceted to address both CM and PV, and highlights the need to prioritize the development of approaches to prevent both types of exposures.

Although interventions targeting the prevention of both CM and PV are lacking, future research could investigate the effectiveness of integrating evidence-based components of interventions from each field. Types of interventions identified as effective for preventing or reducing CM include parent training interventions as well as family-based/multisystemic interventions targeting multiple social systems [41]. Specific effective components include improving parenting practices, parent self-confidence, and attitudes and expectations about parenting, facilitating positive parent–child interactions, providing social-emotional support, and improving child well-being [41]. Evidence-based bullying/PV interventions encompass strategies aimed at developing positive child behaviour, specifically social-emotional skills and skills for positive interactions with peers [42]. Early interventions aimed at younger ages have also been recommended [42]. The Child–Adult Relationship Enhancement (CARE) program is one example of a skills-based and trauma-informed training intervention with promising findings for improving relationships between adults and children/adolescents and facilitating positive behavioural development [43]. Importantly, the CARE program was developed to be used with any adult in any setting [43]. Thus, it is possible that the CARE model could be applied both at home with parents/caregivers and at school with teachers/school staff. Further research is needed to determine if the CARE program is an effective strategy for preventing both CM and PV.

This information is also important for clinicians assessing adolescent patients for mental health problems. It highlights the need for clinicians to consider that their adolescent patients could be experiencing these types of violence (or have experienced them in the past) and the importance of asking about such exposures in an assessment where it is safe and appropriate to do so. Clinicians asking about these experiences should be trained in how to respond to disclosures and be familiar with any mandatory reporting obligations. Prior to asking, the limits of confidentiality should be explained in ways that are age- and developmentally appropriate. While a detailed discussion of response is beyond the scope of this article, the clinician needs to show compassion, and a commitment to supporting the adolescent emotionally and practically, taking into context the type of healthcare that the clinician is providing to the young person (for example, an assessment in the emergency department versus ongoing therapy). For further information, please see the VEGA (Violence, Evidence, Guidance, Action) online education resources [44]. In addition to determining evidence-based approaches to addressing health conditions as part of a treatment plan, it is essential to ask whether such exposures are continuing. This has implications for the safety, health and overall well-being of the adolescent patient; one cannot assume that a reduction in symptoms means that any exposure to CM and/or PV has stopped. Clinicians should also be aware of the increased risk of future health problems when a patient is identified as experiencing CM and/or PV, and of the heightened risk if they have had exposure to both CM and PV.

This research also identifies important sex differences. The prevalence of lifetime CM was higher among female adolescents, consistent with trends observed in a recent systematic review [27], while the prevalence of past-month PV and co-occurring CM and PV was higher among male adolescents. Similar studies have not reported sex differences in the prevalence of co-occurring CM and PV [22, 23]. Sex differences in the prevalence of NSSI, suicidality, mental health disorders, and physical health conditions observed here are also consistent with the literature [45]. Furthermore, assessments of the effect of CM and PV on adolescent health were conducted separately for males and females. Although not directly comparable, the results provide insights into possible sex-specific effects.

The current study also demonstrates a moderating relationship between CM and PV on adolescent mental and physical health, which may differ by sex. In the total sample, exposure to both CM and PV had a multiplicative effect on all outcomes (except any mental health disorder). In sex-stratified models, multiplicative effects were observed for NSSI and suicidal ideation, plans, and attempts for females and on suicidal ideation, suicide attempts, and internalizing and externalizing mental health for males. Specifically, the associations between lifetime CM and NSSI, each suicide outcome, and internalizing and externalizing mental health disorders were greater among those with exposure to past-month PV than those with no PV exposure. Notably, the interaction for physical health (females, total sample) did not show the same multiplicative effect, but rather indicated that the association was greater for those exposed to CM and not PV. These study findings differ from previous research in that very little evidence of interaction effects between CM and PV has been found previously [23]. Overall, these findings provide evidence that exposure to victimization in one setting may depend on other contextual factors. For example, it may be the case that adolescents exposed to both CM and PV have no reprieve from violence since adolescents typically spend most of their time in the settings (e.g., home, school) where this violence may occur. Furthermore, given the pervasiveness of the internet, exposure to cyber-based PV may mean that some adolescents do not have a safe environment even outside of school. While multiplicative effects between CM and PV on suicide-related outcomes were observed for both sexes, multiplicative effects on internalizing and externalizing disorders were noted for males only. This finding has not previously been observed in the literature. Unfortunately, it is not possible to determine with these data why this potential sex difference was noted. It will be important for future research to further evaluate sex and/or gender differences and underlying mechanisms when examining the interaction effects of CM and PV co-occurrence on adolescent health.

This research has important strengths, including the assessment of both CM and PV exposure in a large, representative sample of adolescents; however, the following limitations should be considered. First, causality cannot be inferred from the observed associations since these data are cross-sectional. In addition, the timeframes of each measure vary from ‘ever’ to ‘past month’ and there is overlap in the timeframes of independent and dependent variables. Although we examined mental and physical health as dependent variables, it may also be the case that internalizing symptoms, externalizing symptoms, and physical health conditions are risk factors for victimization [46, 47]. Second, recall bias and social desirability bias may be present due to both the sensitive nature of the questions examined and that the data were gathered with retrospective self-report measures. However, strengths of this study include that this survey section was self-administered, which may have provided the privacy to respond honestly, and the recall time was short since the respondents are adolescents. Third, since a measure of gender identity was unavailable, we could only examine sex differences. Future research should include gender identity to determine if differences exist, including for non-binary individuals. Fourth, the interaction term for suicide plans (males) was not reported due to small cell counts. In addition, the cell counts for the plots for sex-stratified interaction terms did not meet Statistics Canada requirements for data to be vetted and released. Therefore, sex-stratified plots for interactions could be described, but were not provided. Fifth, cumulative effects could not be stratified by sex due to insufficient power. Sixth, the assessment of CM did not include emotional neglect. Seventh, the questions about suicidal plans and attempts were only asked of adolescents who responded that they had thought about suicide within the past year, and thus, depended on the response to suicidal ideation. Finally, it was not possible to examine individual CM and PV types separately.

Extant research indicates that CM and PV are risk factors for the same adverse health outcomes, yet little is known about the combined impact on adolescent health. The current study demonstrates that the cumulative effect of CM and PV exposure heightens odds of mental health problems in adolescence, and may have life-threatening consequences associated with increased odds of suicide thoughts and behaviour. These cumulative effects were also found to be greater compared to experiencing CM or PV alone. Furthermore, interaction effects indicate that experiencing PV may be more harmful in the context of previous or co-occurring CM exposure, and may differ by sex. These findings indicate the need for multifaceted public health strategies that target victimization exposure across multiple contexts. Efforts are needed to effectively prevent victimization occurring both within families and involving peers. Primary prevention of CM and PV remains a priority. Evidence-based guidance is available to help clinicians respond to CM [44]. Health care professionals working with adolescents should be aware of the cumulative and interaction effects associated with experiencing CM and PV, and take this into account when asking about symptoms and conditions, as well as adversities. Future research needs to identify protective factors that may reduce the likelihood of these detrimental health outcomes and further evaluate sex and gender differences in these relationships.

## Supplementary Information

**Additional file 1:** **Supplementary Table 1.**Coefficients and confidence intervals for the interaction between CM and PV on NSSI, suicidality, mental health disorders, and physical health conditions by sex and in the total sample (Model 4).

## Data Availability

The data that support the findings of this study are available from Statistics Canada but restrictions apply to the availability of these data, which were used under license for the current study, and so are not publicly available. Data are however available from the authors upon reasonable request and with permission of Statistics Canada.

## Abbreviations

ACE: Adverse childhood experiences
CD: Conduct disorder
CEVQ: Childhood Experiences of Violence Questionnaire
CM: Child maltreatment
EIPV: Exposure to intimate partner violence
GAD: Generalized anxiety disorder
MDD: Major depressive disorder
NSSI: Non-suicidal self-injury
OCHS: Ontario Child Health Study
OCHS-EBS: Ontario Child Health Study Emotional Behavioural Scales
ODD: Oppositional defiant disorder
PV: Peer victimization
SAD: Separation anxiety disorder
SP: Social phobia
