# Supplementary material for: Adolescent health outcomes: associations with child maltreatment and peer victimization
Source: BMC Public Health. 2022 May 6;22:905. doi: 10.1186/s12889-022-13310-w (PMC9074223; doi:10.1186/s12889-022-13310-w)
Supplement: Supplementary file 1 — Additional file 1: Supplementary Table 1.Coefficients and confidence intervals for the interaction between CM and PV on NSSI, suicidality, mental health disorders, and physical health conditions by sex and in the total sample (Model 4). [file 12889_2022_13310_MOESM1_ESM.docx]

**Supplementary Table 1.** Coefficients and confidence intervals for the interaction between CM and PV on NSSI, suicidality, mental health disorders, and physical health conditions by sex and in the total sample (Model 4)

|  | NSSI | Suicidal Ideation | Suicidal Plans | Suicide Attempts | Any Internalizing Disorder | Any Externalizing Disorder | Any MH Disorder | Any PH Condition |
| --- | --- | --- | --- | --- | --- | --- | --- | --- |
|  | AOR  (95% CI) | AOR  (95% CI) | AOR  (95% CI) | AOR  (95% CI) | AOR  (95% CI) | AOR  (95% CI) | AOR  (95% CI) | AOR  (95% CI) |
| **Female Adolescents** |  |  |  |  |  |  |  |  |
| CM | 3.25 (2.89, 3.64) | 4.41 (3.85, 5.03) | 7.11 (5.53, 9.13) | 7.12 (5.50, 9.22) | 2.70 (2.20, 3.32) | 6.68 (5.37, 8.32) | 3.00 (2.52, 3.57) | 2.31 (2.07, 2.58) |
| PV | 3.58 (3.02, 4.24) | 3.82 (3.23, 4.52) | 5.82 (4.25, 7.98) | 6.08 (4.43, 8.35) | 6.80 (5.55, 8.32) | 8.15 (5.80, 11.46) | 6.77 (5.59, 8.19) | 1.70 (1.50, 1.93) |
| CM x PV | 0.53 (0.43, 0.65) | 0.71 (0.56, 0.91) | 0.38 (0.25, 0.56) | 0.39 (0.27, 0.57) | 0.90 (0.68, 1.19) | 0.89 (0.61, 1.31) | 1.00 (0.78, 1.28) | 0.41 (0.34, 0.50) |
| **Male Adolescents** |  |  |  |  |  |  |  |  |
| CM | 2.05 (1.54, 2.73) | 6.35 (5.11, 7.88) | NR | 5.74 (4.29, 7.70) | 2.83 (2.04, 3.91) | 0.79 (0.58, 1.09) | 1.93 (1.47, 2.51) | 1.13 (1.01, 1.27) |
| PV | 1.83 (1.42, 2.36) | 3.69 (2.86, 4.76) | NR | 3.29 (2.15, 5.03) | 11.98 (8.92, 16.11) | 3.17 (2.43, 4.15) | 9.04 (7.16, 11.42) | 1.15 (1.01, 1.31) |
| CM x PV | 0.88 (0.59, 1.32) | 0.37 (0.26, 0.53) | NR | 0.45 (0.26, 0.77) | 0.58 (0.36, 0.93) | 3.32 (2.02, 5.46) | 1.11 (0.77, 1.61) | 0.93 (0.74, 1.16) |
| **Total Sample** |  |  |  |  |  |  |  |  |
| CM | 2.84 (2.55, 3.17) | 4.97 (4.38, 5.63) | 7.43 (6.07, 9.10) | 6.96 (5.68, 8.53) | 2.82 (2.36, 3.37) | 2.33 (1.97, 2.76) | 2.55 (2.20, 2.95) | 1.68 (1.56, 1.82) |
| PV | 2.66 (2.29, 3.09) | 3.55 (3.06, 4.13) | 6.94 (5.43, 8.87) | 4.59 (3.53, 5.98) | 8.77 (7.37, 10.44) | 4.83 (3.84, 6.06) | 8.03 (6.93, 9.30) | 1.37 (1.25, 1.50) |
| CM x PV | 0.77 (0.63, 0.94) | 0.63 (0.51, 0.77) | 0.24 (0.18, 0.33) | 0.46 (0.34, 0.63) | 0.64 (0.50, 0.81) | 1.56 (1.19, 2.05) | 0.87 (0.71, 1.06) | 0.61 (0.53, 0.70) |
| Note: The coefficient for the interaction term is a ratio of the AOR.  Abbreviations: AOR = odds ratio adjusted for age, ethnicity, household income, single-parent household, and urbanicity; CI = confidence interval; CM = child maltreatment; MH = mental health; NSSI = non-suicidal self-injury; PH = physical health; PV = peer victimization; NR = not reported due to low cell counts. | | | | | | | | |
